# The iTRAQ-based chloroplast proteomic analysis of *Triticum aestivum* L. leaves subjected to drought stress and 5-aminolevulinic acid alleviation reveals several proteins involved in the protection of photosynthesis

**DOI:** 10.1186/s12870-020-2297-6

**Published:** 2020-03-04

**Authors:** Yuexia Wang, Xiaoyan Li, Nana Liu, Shimei Wei, Jianan Wang, Fujun Qin, Biao Suo

**Affiliations:** 1 College of Life Sciences, Henan Agricultural University, No. 63, Nongye Rd., Zhengzhou, 450002 Henan Province China; 2 College of Science, China Agricultural University, Beijing, 100193 China; 3 Department of Pathology, University of Virginia, Charlottesville, VA 22908 USA; 4 College of Food Science and Technology, Henan Agricultural University, No. 63, Nongye Rd., Zhengzhou, 450002 Henan Province China

**Keywords:** Chloroplast proteomics, iTRAQ, Drought, Wheat, Reactive oxygen species

## Abstract

**Backgrounds:**

The perturbance of chloroplast proteins is a major cause of photosynthesis inhibition under drought stress. The exogenous application of 5-aminolevulinic acid (ALA) mitigates the damage caused by drought stress, protecting plant growth and development, but the regulatory mechanism behind this process remains obscure.

**Results:**

Wheat seedlings were drought treated, and the iTRAQ-based proteomic approach was employed to assess the difference in chloroplast protein content caused by exogenous ALA. A total of 9499 peptides, which could be classified into 2442 protein groups, were identified with ≤0.01 FDR. Moreover, the contents of 87 chloroplast proteins was changed by drought stress alone compared to that of the drought-free control, while the contents of 469 was changed by exogenous ALA application under drought stress compared to that of drought stress alone. The Gene Ontology (GO) annotation and Kyoto Encyclopedia of Genes and Genomes (KEGG) analysis results suggested that the ALA pretreatment adjusted some biological pathways, such as metabolic pathways and pathways involved in photosynthesis and ribosomes, to enhance the drought resistance of chloroplasts. Furthermore, the drought-promoted H_2_O_2_ accumulation and O_2_^−^ production in chloroplasts were alleviated by the exogenous pretreatment of ALA, while peroxidase (POD) and glutathione peroxidase (GPX) activities were upregulated, which agreed with the chloroplast proteomic data. We suggested that ALA promoted reactive oxygen species (ROS) scavenging in chloroplasts by regulating enzymatic processes.

**Conclusions:**

Our results from chloroplast proteomics extend the understanding of the mechanisms employed by exogenous ALA to defend against drought stress in wheat.

## Background

Wheat (*Triticum aestivum* L.) is one of the most important crops worldwide. However, wheat plants often suffer from drought stress throughout the whole growth stage, which causes serious physiological and biochemical damage, and as a result, the yield is reduced [1]. Plant chloroplasts are one of the most sensitive components to drought stress. Drought causes deteriorative changes in the structure of the subunits within the chloroplast, such as the thylakoid and granum [2], thus disrupting the photosynthetic function of the chloroplast. Under drought conditions, wheat plants cannot sustain a normal performance to provide sufficient photosynthetic products to the plant unless the photosynthetic functions are adequately protected [3].

Sustaining plant chloroplast photosynthetic function under drought stress requires the regulation of chloroplast proteins. In reports from past decades, some proteins that sustain plant chloroplast photosynthetic function under drought stress have been characterized. For example, the content and activity of antioxidant enzymes increase significantly under drought stress, which is aimed at scavenging the reactive oxygen that is produced [4]. Alternative oxidase has been proven to participate in a respiratory electron transport chain pathway that is essential for maintaining photosynthetic performance during drought stress [5]. Limited reports have shown that the translation, phosphorylation, and translocation of chloroplast protein play an important role in the sustainment of photosynthetic function under drought conditions.

However, the functional regulation of chloroplast proteins is inexplicable by using a semiautonomous analytical method. Among the 2000–3000 kinds of proteins in plant chloroplasts, only approximately 100 are assembled in chloroplasts, and the others are encoded by nuclear genes. They are then translated in the cytoplasm and are finally transported to chloroplasts [6]. Therefore, whole leaf-based studies have a limitation in attempting to reveal the function of chloroplast proteins. A proteomic analysis following chloroplast isolation provides a promising approach for elucidating chloroplast-located protein regulation and its relationship with stress resistance [7, 8]. Until now, the strategy of chloroplast proteomics has been employed to investigate the possible molecular regulatory pathways in response to biotic or abiotic stress in a variety of plants, such as the chlorosis mechanisms of *Nicotiana tabacum* leaves induced by viral infection [9], the response mechanism of tomato (*Solanum lycopersicum* L.) to drought stress and recovery [10], and the pathways associated with salt tolerance in *Kandelia candel* [11]. However, to the best of our knowledge, few studies have reported on the chloroplast proteomic analysis of wheat in response to drought stress, especially when an exogenous regulator is applied.

Growth regulatory substances have long been considered promising for improving the resistance of plants to environmental stress [12]. 5-aminolevulinic acid (ALA) is a precursor of porphyrins in plant chloroplasts [13]. In recent decades, the exogenous application of ALA at low concentrations has exhibited a protective effect on plant development in response to a variety of stresses, such as NaCl [14], heavy metal [15], waterlogging [16], photodynamical [13], and chilling [17] stresses. Under drought stress, the exogenous application of ALA has been proven to alleviate the damage, which was shown in reports on Kentucky bluegrass [18], alfalfa (*Medicago varia* Martyn.) [19], *Leymus chinensis* [20], canola (*Brassica napus* L.) [21], and wheat [22, 23]. For wheat, the use of ALA is more attractive because this substance was reported to promote growth and alleviate the yield loss caused by dry conditions [24].

Despite the large number of reports on the response of crops to drought, little information is currently available on the plant ALA-induced alleviation mechanism. Because of the predominantly protective effect of ALA against environmental stress, its mechanism has increasingly gained attention. According to the existing evidence, the exogenous application of ALA increases the chlorophyll content, activates antioxidant enzymes, reduces lipid peroxidation of the membrane [13], promotes photosynthesis [15], enhances the tetrapyrrole biosynthetic pathway and proline accumulation [14], improves the metabolism of polyamines [17], regulates nitrogen metabolism, absorption, aggregation and distribution of nutrient ions [22, 25], inhibits the microstructural damage in chloroplasts by increasing the proportion of intact thylakoids [26], and regulates the expression of genes [27]. Our previous study showed that ALA pretreatment enhanced the photosynthetic capacity of drought-stressed wheat seedlings [23], but the regulatory mechanism behind this effect is still unknown.

In this study, we used the iTRAQ technique to identify differentially abundant proteins in the chloroplasts of wheat seedlings under drought stress that were pretreated with exogenous ALA. The aim of this study was to explore the major proteins and enzymes involved in chloroplast regulation in response to drought stress. We also provided a theoretical basis for understanding the regulatory pathways of exogenous ALA when the plant is subjected to drought stress.

## Results

### Effects of exogenous ALA on the physiological response of wheat under drought stress

**Fig. 1:**
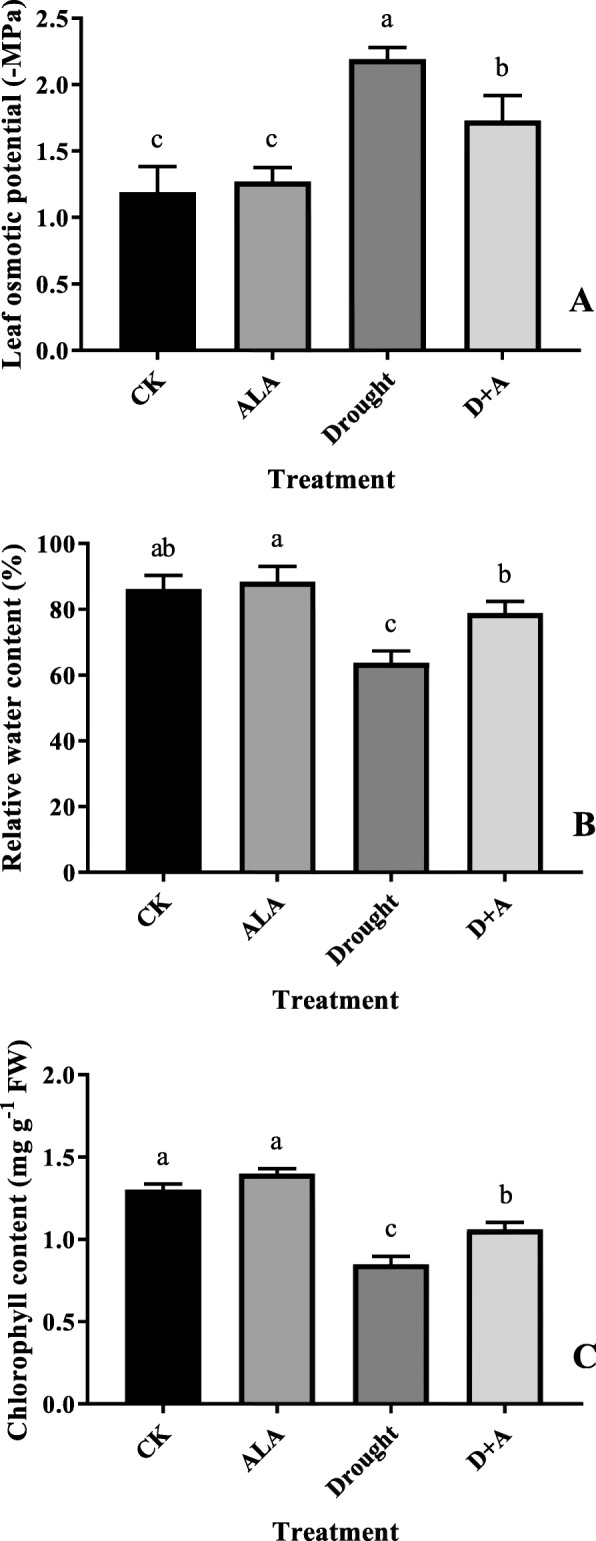
Effects of ALA on leaf osmotic potential (**a**), relative water content (**b**), and chlorophyll content (**c**) in wheat seedlings under drought stress. Each value is the mean of three replicates, and the vertical bars indicate the standard errors (SE). CK, treated with 0 mg L^− 1^ ALA + distilled water; ALA, treated with 100 mg L^− 1^ ALA and distilled water; drought, treated with 0 mg L^− 1^ ALA without distilled water; D + A, treated with 100 mg L^− 1^ ALA without distilled water. The wheat plants underwent drought stress 3 d after pretreatment with or without ALA

In this study, the mitigative effect of exogenous ALA on the drought response of wheat was evaluated by measuring the physiological traits of wheat. As shown in Fig. 1, drought stress significantly increased the leaf osmotic potential, but decreased the RWC and chlorophyll contents (*P* < 0.05). However, when exogenous ALA was pre-treated on wheat leaves, the osmotic potential decreased by 21% compared to that under drought stress alone, while the RWC and chlorophyll contents increased by 24 and 25%, respectively. These three indicators formed the basis of the physiological response analysis, which proposed that the exogenous ALA could alleviate the response due to drought stress. Therefore, leaf samples obtained under different treatments, which were assumed to have different chloroplast proteomes, were used for the proteomics analysis.

### Primary data analysis and protein identification

The chloroplast proteomics were analyzed in three biological triplicates of leaves of wheat seedlings under four treatments. After LC-MS determination and bioinformatic analysis, a total of 9499 peptides were identified with ≤0.01 FDR (Supplementary Table 1) and could be classified into 2442 protein groups (Supplementary Table 2). Principal component analysis (PCA) showed the similarities or difference of samples among CK (normal watering), drought (without water supplement), ALA (normal watering with exogenous ALA application), and D + A (without water supplement but with exogenous ALA application) (Supplementary Figure 1). The total variances in the proteins of the four samples were 44.4% (PC1) and 20% (PC2) with an acceptable separation indicated by the four different clusters. The D + A treated sample were the farthest to the CK sample compare with ALA and drought treated ones. This indicated that exogenous ALA application under drought stress could cause most serious proteomic changes, compared to drought or ALA treated alone.

**Fig. 2:**
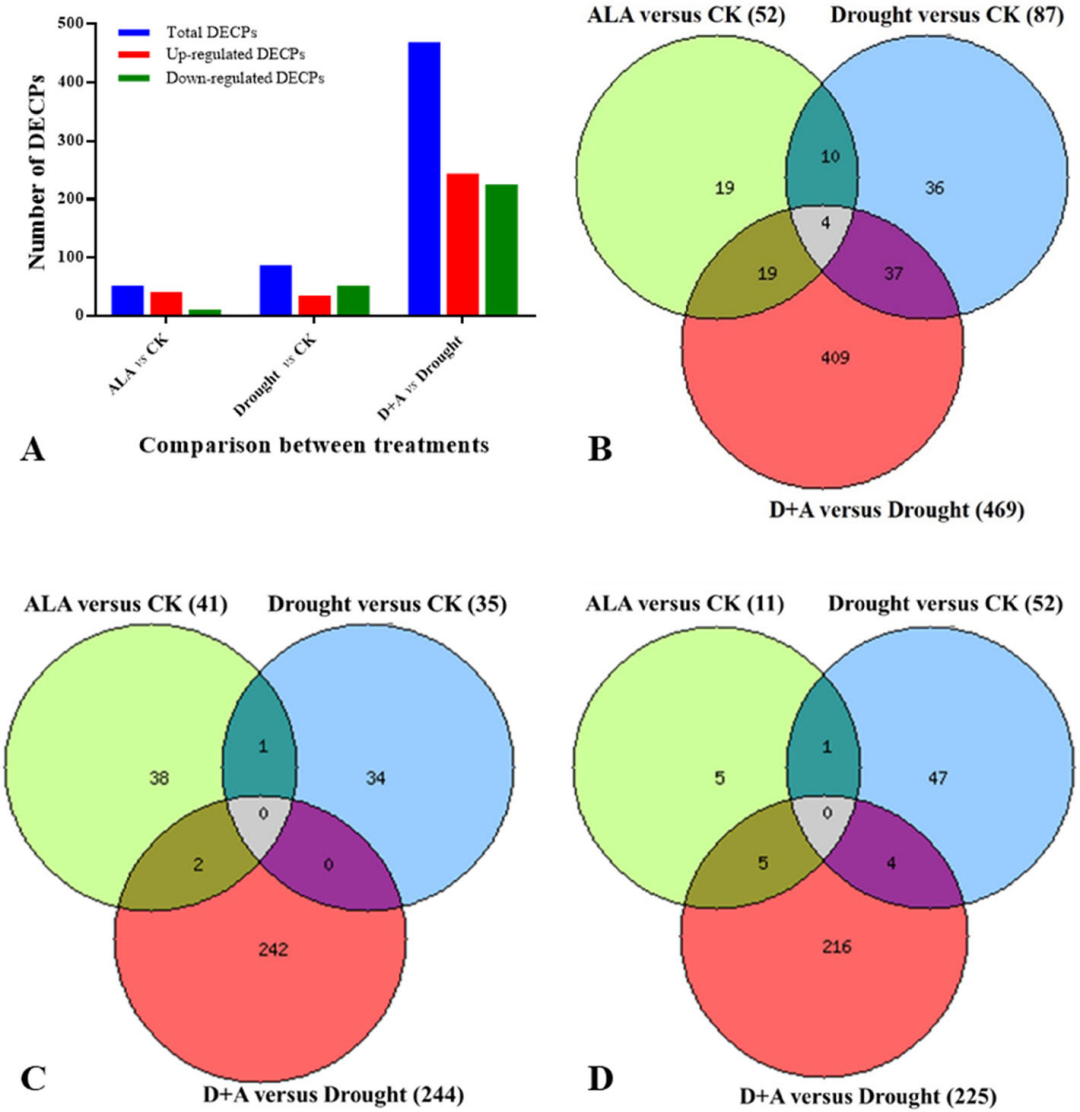
Venn diagrams of the differentially abundant chloroplast proteins (DACPs) in wheat leaves between different treatments. **a** Total DACPs; **b** Upregulated DACPs; **c** Downregulated DACPs. CK, treated with 0 mg L^− 1^ ALA + distilled water; ALA, treated with 100 mg L^− 1^ ALA and distilled water; drought, treated with 0 mg L^− 1^ ALA without distilled water; D + A, treated with 100 mg L^− 1^ ALA without distilled water. The wheat plants underwent drought stress 3 d after pretreatment with or without ALA

**Fig. 3:**
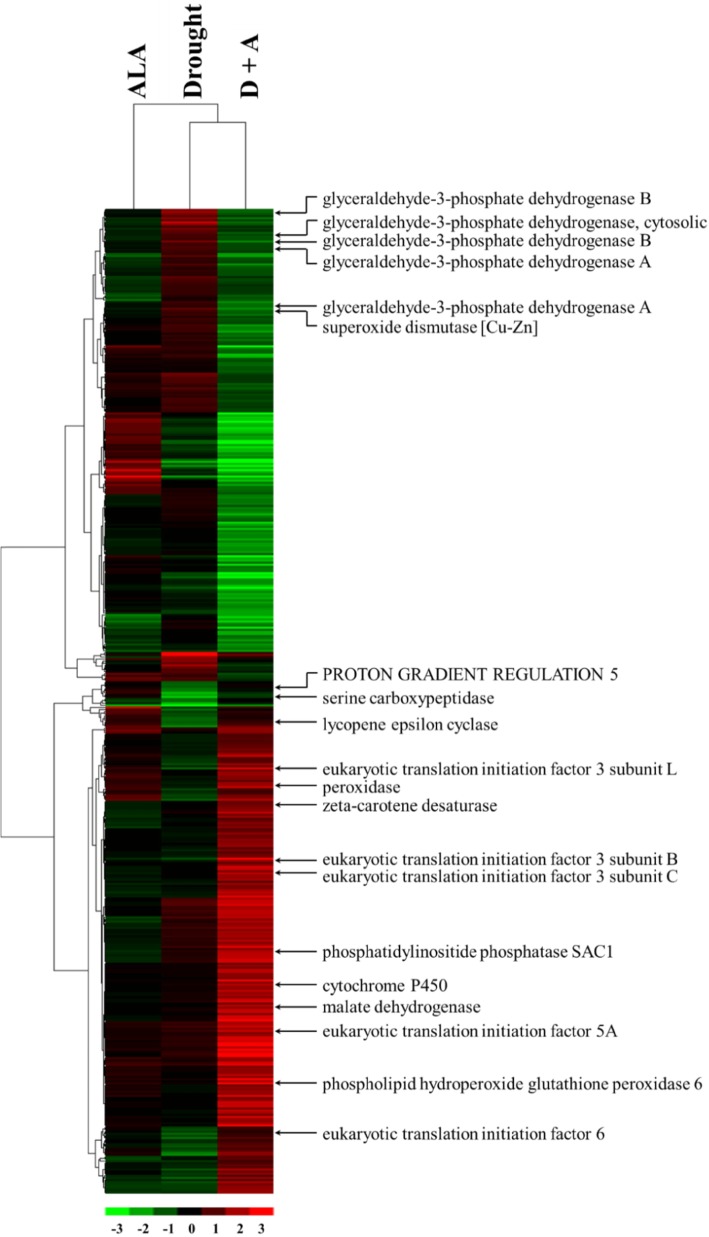
Hierarchical clustering analysis of 469 differentially abundant chloroplast proteins (DACPs) in D + A versus drought. The content patterns of the proteins were hierarchically clustered based on the ratio as a log_2_ scale. Each row in the color heat map indicates a single chloroplast protein extracted from ALA (treated with 100 mg L^− 1^ ALA and distilled water), drought (treated with 0 mg L^− 1^ ALA without distilled water), and D + A (treated with 100 mg L^− 1^ ALA without distilled water). The sample from plants treated with distilled water was used as a reference control. The red-colored clusters represent upregulated proteins, and the green-colored clusters represent downregulated proteins

The differentially abundant chloroplast proteins (DACPs) were identified between the ALA treatment and the CK, aiming to understand the possible influence of ALA pretreatment on chloroplast protein accumulation under normal watering conditions. The DACPs between the drought treatment and CK were identified to understand the effect of drought stress on protein accumulation in chloroplasts. The comparison between the ALA plus drought treatment and drought alone was aimed at understanding the possible alleviation mechanism of exogenous ALA on drought-induced damage. As shown in Fig. 2 and Supplementary Table 2, 52, 87, and 469 DACPs were identified between the treatments of ALA and CK, drought and CK, and ALA plus drought and drought alone, respectively. Among the DACPs, 41, 35, and 244 were upregulated, while 11, 52, and 225 were downregulated. The identified proteins were notably different in the ALA, drought alone, and ALA plus drought treatments compared to the control treatment (Fig. 3). These identified proteins were filtered to verify whether the changes in protein abundance are significant. This was based on a fold change ≥1.5 or ≤ 0.667 and *p* ≤ 0.05. The results revealed that the ALA pretreatment under drought stress had a greater influence on chloroplast protein accumulation than did the ALA and drought alone treatments.

qRT-PCR assays were performed to validate the gene expression patterns of six randomly selected DACPs. For each DACP, the gene transcriptions were evaluated under ALA, drought, and D + A treatments, respectively, using the CK as reference sample. A subset of 18 transcriptions was tested to confirm the proteomic results. As shown in Supplementary Figure 2, a strong positive correlation was observed (*R*^2^ = 0.78), confirming the validity of the proteomic data used in this study.

### Functional categorization of the DACPs

**Fig. 4:**
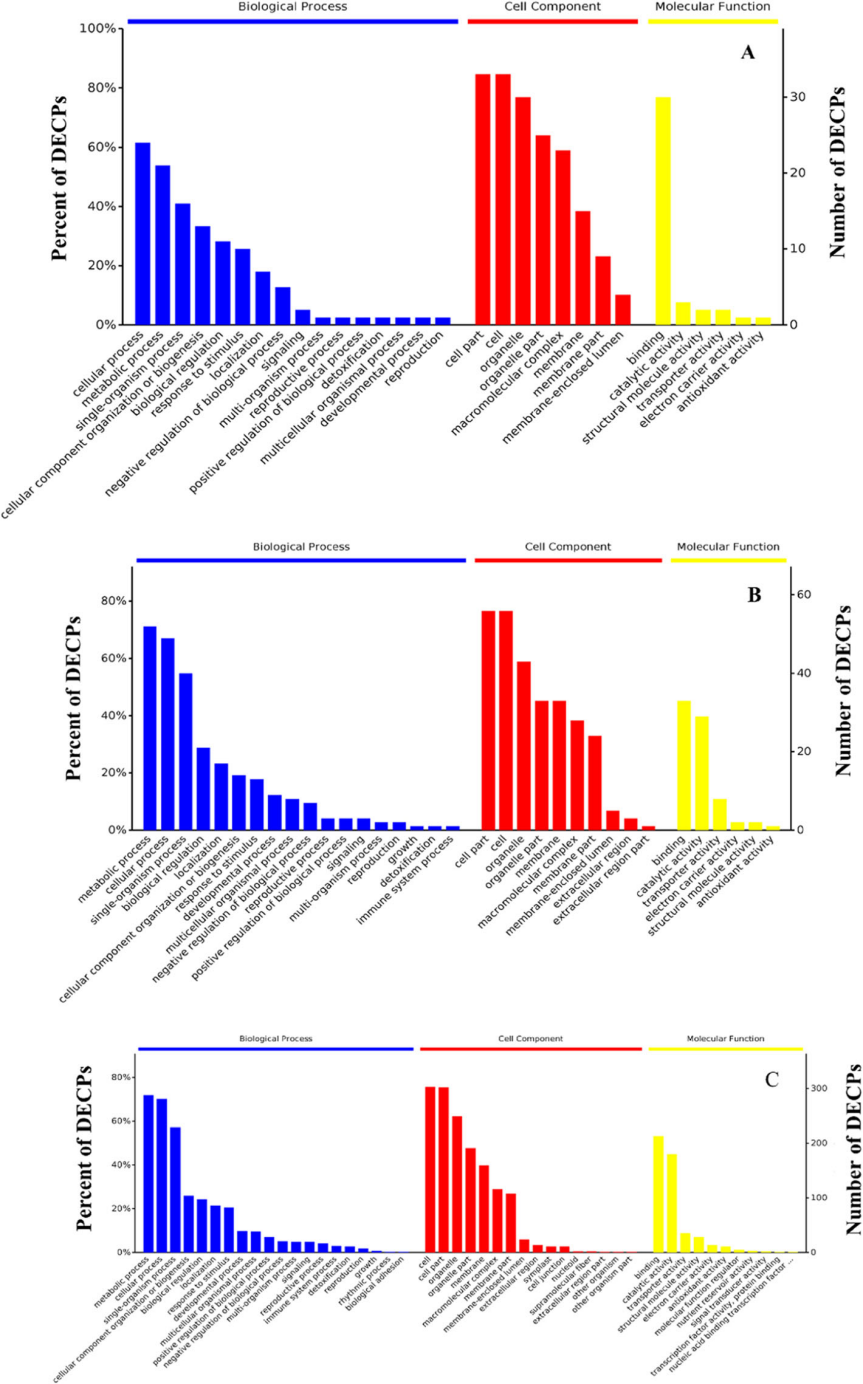
The gene ontology profile of the differentially abundant chloroplast proteins (DACPs) in wheat seedling leaves under drought stress and exogenous ALA pretreatment. The bar charts represent the functional annotations of the DACPs until the second level of complexity in ALA versus CK (**a**), drought versus CK (**b**), and D + A versus drought (**c**). CK, treated with 0 mg L^− 1^ ALA + distilled water; ALA, treated with 100 mg L^− 1^ ALA and distilled water; drought, treated with 0 mg L^− 1^ ALA without distilled water; D + A, treated with 100 mg L^− 1^ ALA without distilled water. The wheat plants underwent drought stress 3 d after pretreatment with or without ALA

To understand the protein functions associated with the DACPs under ALA pretreatment and drought stress, GO analysis was performed using BLAST2GO software. The GO functional categorization generated 327 annotations from the 52 DACPs between ALA-pretreated only wheat seedlings and CK (Fig. 4a). In that study, 116, 39, and 172 annotations could be classified as the first level classifications of biological processes, molecular functions, and cellular components, respectively. Among the biological process classifications, 24 and 21 DACPs were classified into the categories of cellular processes (GO:0009987) and metabolic processes (GO:0008152), respectively. In the cellular component classification, the two main categories were cell part (GO:0044464) and cell (GO:0005623). In the molecular function classification, the most common category was binding (GO:0005488).

The GO functional categorization generated 603 annotations from the 87 DACPs between drought-treated only seedlings and CK (Fig. 4b). In that study, 246, 282, and 75 annotations could be classified as the first level classifications of biological processes, cellular components, and molecular functions, respectively. In the biological process classification, 52 and 49 DACPs were classified into the categories of metabolic processes (GO:0008152) and cellular processes (GO:0009987). In the cellular component classification, the 56 DACPs were classified into both the cell part (GO:0044464) and cell (GO:0005623) categories. Among the molecular function classifications, 33 and 29 DACPs were classified into the categories of binding (GO:0005488) and catalytic activity (GO:0003824).

Regarding GO enrichment for the DACPs in D + A vs. drought alone, 3366 annotations were generated from the 469 DACPs (Fig. 4c). The top five enriched GO terms in the biological process classification were metabolic processes (GO:0008152), cellular processes (GO:0009987), single-organism processes (GO:0044699), cellular component organization (GO:0071840), and biological regulation (GO:0065007). For the cellular component classification, the top five enriched GO terms were cell (GO:0005623), cell part (GO:0044464), organelle (GO:0043226), organelle part (GO:0044422), and membrane (GO:0016020). The most enriched terms within the molecular function classification were binding (GO:0005488) and catalytic activity (GO:0003824).

### KEGG pathway analysis of the DACPs

**Fig. 5:**
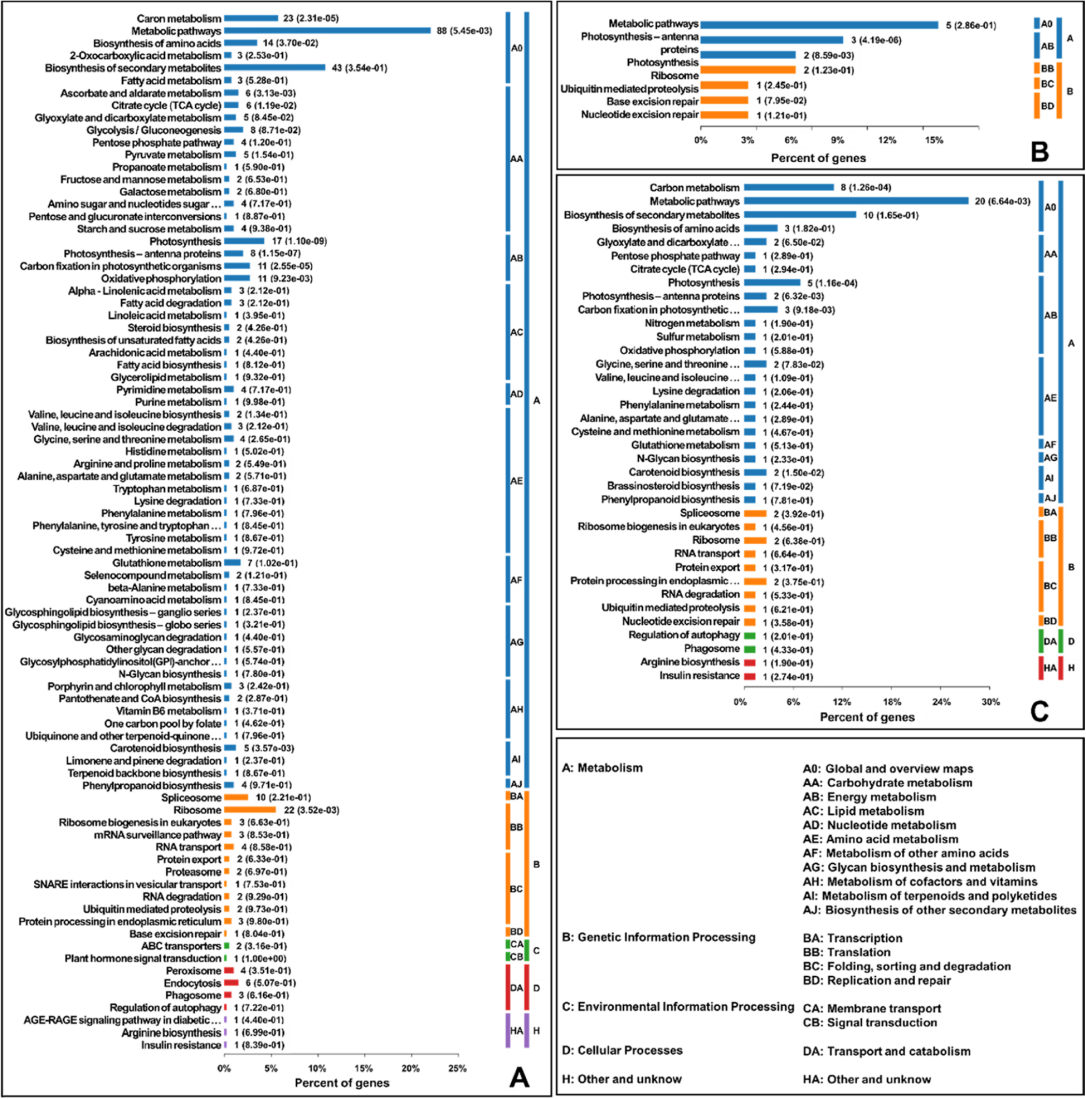
Enriched KEGG pathways of differentially abundant chloroplast proteins (DACPs) in wheat seedling leaves under drought stress and exogenous ALA pretreatment. **a** D + A versus D; **b** ALA versus CK; and **c**, drought versus CK. The number of involved proteins in a specific pathway and corresponding *p*-values are shown on the right side of the column. CK, treated with 0 mg L^− 1^ ALA + distilled water; ALA, treated with 100 mg L^− 1^ ALA and distilled water; drought, treated with 0 mg L^− 1^ ALA without distilled water; D + A, treated with 100 mg L^− 1^ ALA without distilled water

The functions of the DACPs between the different treatments were further analyzed by the KEGG pathway annotation method (Fig. 5). The 469 DACPs between the D + A and drought-treated seedlings were subcategorized into 84 KEGG classifications. “Metabolic pathways” was the most represented pathway, followed by “biosynthesis of secondary metabolites”, “carbon metabolism”, “ribosome”, and “photosynthesis” (Fig. 5a). “Metabolic pathways” was also the most represented pathway in ALA versus CK and drought versus CK (Fig. 5b and c). In ALA versus CK, the four most represented pathways subcategorized by 52 DACPs were metabolic pathways, photosynthesis – antenna proteins, photosynthesis, and ribosomes. The four most represented pathways subcategorized by the 87 DACPs of drought versus CK were metabolic pathways, biosynthesis of secondary metabolites, carbon metabolism, and photosynthesis. The KEGG pathway analysis results indicated that drought stress and exogenous ALA application mainly influence the pathways of metabolites and photosynthesis.

### Protein-protein interactions of DACPs

**Fig. 6:**
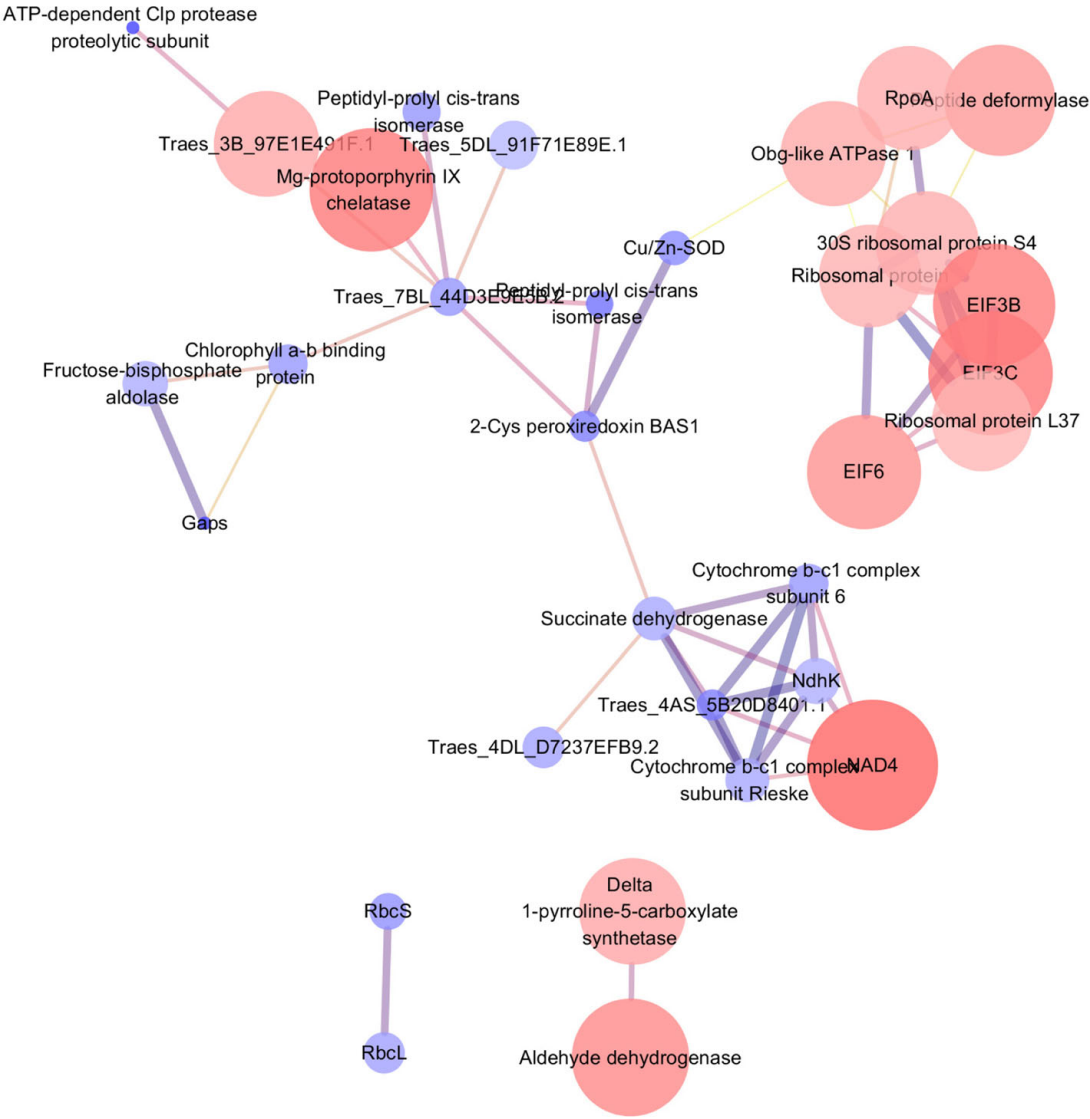
Protein-protein interaction networks of differentially abundant chloroplast proteins (DACPs) of wheat seedlings treated with 100 mg L^− 1^ ALA without distilled water (D + A) compared to that treated by drought stress alone. Proteins are indicated with nodes, and interactions between proteins are represented by edges. The edge colors indicate the combined score. The node colors represent upregulated protein (red) or downregulated protein (blue), and the size of node also indicates the change in the level of protein expression. Nad4, NADH-ubiquinone oxidoreductase chain 4; NdhK, NAD(P)H-quinone oxidoreductase subunit K; RpoA, DNA-directed RNA polymerase subunit alpha; RbcL, Ribulose bisphosphate carboxylase large chain; RbcS, Ribulose bisphosphate carboxylase small chain; Cu/Zn-SOD, superoxide dismutase Cu/Zn isoform; Gaps, glyceraldehyde-3-phosphate dehydrogenase

The DACPs were analyzed using the STRING online search tool, and the PPIs with a score higher than 0.7 were used to build an interaction network by employing Cytoscape software. As shown in Fig. 6, the PPI network of D + A vs. drought alone contained 519 edges, which is higher than the expected number (423). The excessive edge number denoted that the proteins have more PPIs, compared to what would be expected for a random set of proteins of similar size drawn from the genome. The results suggested that the DACPs are at least partially biologically connected as a group.

## Discussion

Plant chloroplast is essential for the sustainment of photosynthetic function under drought condition, which can be attributed to the regulation of protein content. A detailed knowledge of the changes in chloroplast proteins in response to drought is essential to understand the mechanisms underlying stress adaptation. In this study, we have focused on the chloroplast response to drought stress with or without ALA pretreatment and evaluated its possible contribution to drought resistance of photosynthesis.

According to the proteomic data, a cytochrome P450 71A9-like protein (A0A1D5YKA1) was upregulated by ALA pretreatment under drought stress. In *Arabidopsis*, cytochrome P450 mediates the Baeyer-Villiger oxidation of castasterone to brassinolide in brassinosteroid biosynthesis [28]. Brassinosteroid is a polyhydroxylated steroidal plant hormone that plays a pivotal role in regulating various plant growth and development processes. Many studies have already claimed that brassinosteroids improve growth and yield in agricultural systems under various stress conditions, including drought [29]. The novelty of the accumulation of cytochrome P450 in present result was that, the pretreatment with ALA could enhance drought resistance by promoting brassinosteroid biosynthesis, possibly contributing to sustaining the yield of wheat under drought stress [24].

When plant is suffering from drought stress, the carbon/nitrogen ratio is normally altered and induces nutrient mobilization because of the CO_2_ limitation. In the ALA-pretreated seedlings under drought stress, a 1.7-fold increase was observed for malate dehydrogenase (MDH) (A0A1D5YPG2) compared to that of seedling under drought stress alone. MDH plays a key role in the regulation of the carbon/nitrogen ratio by participating in the Krebs cycle. When CO_2_ assimilation is restricted under environmental stresses, the accumulation of MDH in chloroplasts accompanies with the regeneration of the electron acceptor NADP^+^ [30], which is beneficial for the short-term adjustment of the stromal NADPH redox stage [31]. The observed downregulation of the biosynthesis of amino acid-related proteins by ALA pretreatment is suggestive of stimulating the alternative catabolic pathways to generate energy in need to preserve its existing energy [32].

It is known that there are at least two photosynthetic cyclic electron transport (CET) pathways in most C3 plants: the NAD(P) H dehydrogenase (NDH)-dependent pathway and the proton gradient regulation5 (PGR5) (H9C8A5)-dependent pathway [33]. In the present study, when wheat seedlings were pretreated with ALA, NAD(P) H dehydrogenase was significantly downregulated under drought stress. However, the key protein in the ferredoxin-dependent CET pathway, proton gradient regulation 5 (PGR5), was downregulated under drought stress. Interestingly, PGR5 accumulated significantly with the pretreatment of ALA. In addition, ribulose bisphosphate carboxylase (small and large chain) was downregulated in the ALA-pretreated wheat seedlings, but ribulose bisphosphate carboxylase/oxygenase activase showed a 1.9-fold increase. In both *Arabidopsis thaliana* and *Chlamydomonas reinhardtii*, PGR5-mediated CEF and showed that PGR5/PGRL1-Fd CEF functions in accordance with an ATP/redox control model [33]. The *Arabidopsis* plants overexpressing PGR5 exhibited better survival when exposed to high light and drought stress than did the wild-type plants [34]. Therefore, the present proteomic data indicated that exogenous ALA application protected wheat seedlings under drought stress by activating a PGR5-dependent pathway.

The serine carboxypeptidase-like protein family is located on the cytomembrane and has been shown to play a key role in plant growth, development and stress responses [35, 36]. These proteins in rice leaves was significantly accumulated after treatments with benzothiadiazole, salicylic acid, jasmonic acid and 1-amino cyclopropane-1-carboxylic acid, etc. The over-abundant plants also showed an increased tolerance to oxidative stress and upregulated expression of oxidative stress-related genes [37]. The present proteomic data showed that serine carboxypeptidase (A0A1D6ANR8) was downregulated under drought stress alone but accumulated by 2.1-fold when ALA was applied prior to drought stress. The upregulation effect of exogenous ALA was not significantly shown in the drought-free condition. The results suggested that serine carboxypeptidase may be involved in the regulation of exogenous ALA to protect against drought stress.

**Fig. 7:**
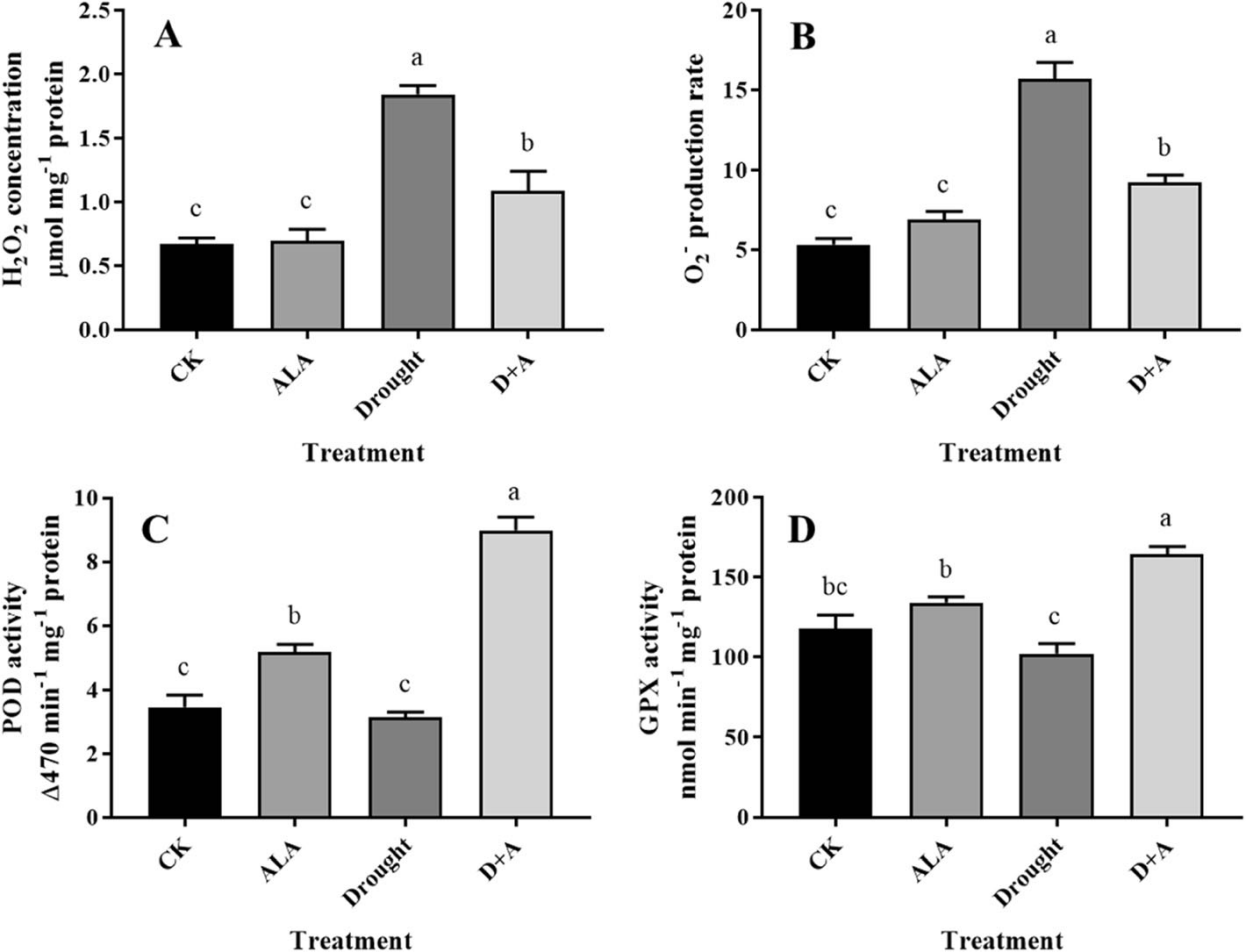
Effects of exogenous ALA on the H_2_O_2_ concentration (**a**), O_2_^−^ production rate (**b**), POD (**c**) and GPX (**d**) activities in chloroplasts in leaves of wheat seedlings exposed to drought stress. CK, treated with 0 mg L^− 1^ ALA + distilled water; ALA, treated with 100 mg L^− 1^ ALA and distilled water; drought, treated with 0 mg L^− 1^ ALA without distilled water; D + A, treated with 100 mg L^− 1^ ALA without distilled water. Error bars represent SD values (*n* = 3). Different letters indicate significant differences among the treatments (*P* < 0.01)

A number of recessive resistance genes have been shown to encode eukaryotic translation initiation factors (EIFs). A core set of EIFs is conserved to facilitate the assembly of a translation-competent ribosome at the initiation codon of a mRNA [38]. An increasing number of studies have shown that plant EIFs play roles in stress resistance, such as *eiF (iso)4E* in *B. rapa* [39] and *eif2B-beta* in *B. juncea* [40] in response to TuMV. In the present study, two isoforms of EIF, 6 and 3 L, were downregulated in response to drought stress, while five isoforms of EIF, 5A, 6, 3B, 3C, and 3 L, accumulated in the drought-stressed wheat seedlings pretreated with exogenous ALA. Eukaryotic translation initiation is a highly regulated and complex stage of gene translation, which requires the action of at least 13 core initiation factors and five auxiliary factors [38, 41]. Among the EIFs, EIF 5A is a highly conserved protein in all eukaryotic organisms, and the mutant analysis in *Arabidopsis* has demonstrated that it influences Cd sensitivity by affecting Cd uptake, accumulation, and detoxification [42]. The accumulation of these wheat EIFs under drought stress represents new targets and mechanisms for the protective strategies of the exogenous application of ALA. It is known that oxidative stress often occurs as a result of drought stress. As shown in Fig. 7a and b, the H_2_O_2_ content and O_2_^−^ production rate in the wheat chloroplasts both increased significantly in response to drought stress (*P* < 0.01). The ALA pretreatment alleviated the damage caused by reactive oxygen species (ROS) in chloroplasts, as shown by the fact that both the H_2_O_2_ content and O_2_^−^ production rate noticeable decreased, although they were still higher than those of the drought-free seedlings. In recent decades, studies have shown that the chemical nature of reactive oxygen species (ROS) indicates that they are not only potentially harmful to plant cells but that they also act as a second messenger in signal transduction in response to environmental stress [43]. According to the present chloroplast proteomic data, phospholipid hydroperoxide glutathione peroxidase (GPX) (A0A1D5U2X7) and peroxidase (POD) (A0A1D6BMX2) were 1.7- and 2.2-fold upregulated by ALA pretreatment, respectively, compared to those of drought alone. The proteomic data were coincident with the results from the enzymatic analysis of GPX and POD in the wheat chloroplasts (Fig. 7c and d). However, the superoxide dismutase Cu/Zn isoform (Cu/Zn-SOD) (C3VQ50) was 0.5-fold downregulated, and catalase (CAT) (A0A0A7MA13) did not show a significant change (*P* < 0.05) in either drought alone or ALA plus the drought treatment. The results could be attributed to the fact that Cu/Zn-SOD mainly functions as a ROS scavenger in the cytoplast and only scavenges ROS a little in chloroplasts [44]. GPX catalyzes the reduction of H_2_O_2_, organic hydroperoxide, and lipid peroxides using glutathione (GSH), thioredoxin (TRX) and/or other reducing equivalents and is generally considered to be the main line of enzymatic defense against oxidative membrane damage [45–47]. GPX has been proven to function as both a redox transducer and an ROS scavenger in abscisic acid and drought stress response in *Arabidopsis* [48]. The over-accumulation of GPX in the chloroplasts of transgenic tobacco removed unsaturated fatty acid hydroperoxides that were generated in cellular membranes under stress conditions, leading to the maintenance of membrane integrity and increased tolerance to oxidative stress caused by various stress conditions [49]. PODs are primarily localized in cell walls and vacuoles [50], but a recent report has shown that PODs are also present in chloroplasts [51]. POD catalyzes oxidative reactions by converting hydrogen peroxide (H_2_O_2_) to H_2_O, while a substrate is being oxidized, thus playing an important role in drought resistance [52]. Phosphoinositide phosphatase SAC8 is also involved in reducing the accumulation of ROS [53]. In our study, the proteomic data showed that the exogenous pretreatment of ALA increased the level of the phosphoinositide phosphatase SAC8 isoform SAC1 (A0A1D5SX72) in wheat chloroplasts, which was similar to the result of NaHS pretreatment in drought-stressed wheat seedlings [54]. In the present study, the exogenous ALA-triggered accumulations of GPX, POD, and phosphoinositide phosphatase SAC1 indicated were ROS scavengers when the wheat plants were suffering from drought stress.

In higher plant leaves, there are three kind of glyceraldehyde-3-phosphate dehydrogenase (Gaps) that participate in the environmental stress response by catalyzing key steps in energy and reducing power partitioning in cells; GapA and GapB are in chloroplasts, while GapC is in the cytoplasm [55, 56]. According to the proteomic data, GapB (A0A1D5XJY0, A0A1D5YL67) accumulated when the wheat seedlings were subjected to drought stress alone, while GapA (A0A1D5T798, A0A1D5TTT4), GapB, and GapC (A0A1D6C9L9) all showed significant downregulation when exogenous ALA was used as a pretreatment. The accumulation of Gap benefits the transduction of the ROS hydrogen peroxide (H_2_O_2_) signal in response to drought stress, and a knockout of Gap decreases stomatal sensitivity to ABA and renders plants less responsive to water deficits than the wild type [57]. When wheat seedlings were pretreated with ALA, the downregulation of Gaps was coordinated with a lower content of H_2_O_2_ in a previous report [23], which was most likely associated with the accumulation of the ROS scavengers GPX and POD.

There are also some nonenzymatic molecular antioxidants that participate in ROS scavenging, including ascorbate, α-tocopherol, carotenoids, and glutathione [58]. The lycopene epsilon cyclase negatively regulates carotenoid synthesis by the β-branch-specific pathway during the stress response [59]. The lycopene epsilon cyclase (C1IP74) was down represented in response to drought stress, which contributed to sustaining the content of carotenoids at a certain level to scavenge ROS in chloroplasts [60]. The exogenous pretreatment of ALA accumulated lycopene epsilon cyclase in chloroplasts under drought stress, implying that a high carotenoid content was not yet necessary because of the lower ROS production and higher chlorophyll content when the drought-stressed wheat seedlings were pre-treated with exogenous ALA than when they were treated with drought stress alone. Zeta-carotene desaturase (A0A1D5U7A6) is another chloroplast protein that is essential for carotenoid biosynthesis. It was upregulated in the ALA-pretreated wheat seedlings compared to in the seedlings under drought stress alone. Furthermore, the accumulation of zeta-carotene desaturase plays an important role in chlorophyll biosynthesis, coloration, chloroplast biogenesis, and PSII capacity [61]. The results suggested that pretreatment with ALA participated in the nonenzymatic antioxidant process by regulating carotenoid biosynthesis-related proteins.

Among the identified DACPs, there were some non-chloroplastic proteins identified and showed significant changes under treatments. For example, the histones H2A, H2B, H3, and H4 were all down-regulated by the exogenous application of ALA under drought stress, which were coincident with results reported in rice under cold stress [62]. The non-chloroplast protein contaminations should be attributed to the Percoll-based chloroplast purification protocol that cannot fully exclude cross-contaminations originating from other plastids and cell compartments, even it has been well established [7, 8]. Histones are prone to reversible post-translational modifications such as phosphorylation, ubiquitination, acetylation, methylation, and glycosylation, which allow the proteins to respond flexibly to stimuli [63]. They are DNA-binding proteins of nucleoids or nucleomorph, while may possibly be post-translationally modified and perform the role in chloroplasts [64, 65].

## Conclusions

In summary, an iTRAQ-based proteomics approach was employed to assess the changes in chloroplast protein content caused by exogenous ALA in wheat leaves under drought stress. A total of 9499 peptides were identified with ≤0.01 FDR and could be classified into 2442 protein groups. Moreover, the contents of 87 chloroplast proteins were changed by drought stress alone, while 469 were changed by exogenous ALA application under drought stress. The results suggest that ALA pretreatment changed some biological pathways, such as metabolic pathways and pathways involved in photosynthesis and ribosomes, to enhance the drought resistance of chloroplasts. Moreover, ALA promoted ROS scavenging by regulating enzymatic processes. Our results from chloroplast proteomics extend the understanding of the mechanisms employed by exogenous ALA to defend against drought stress in wheat.

## Methods

### Plant materials and treatments

**Fig. 8:**
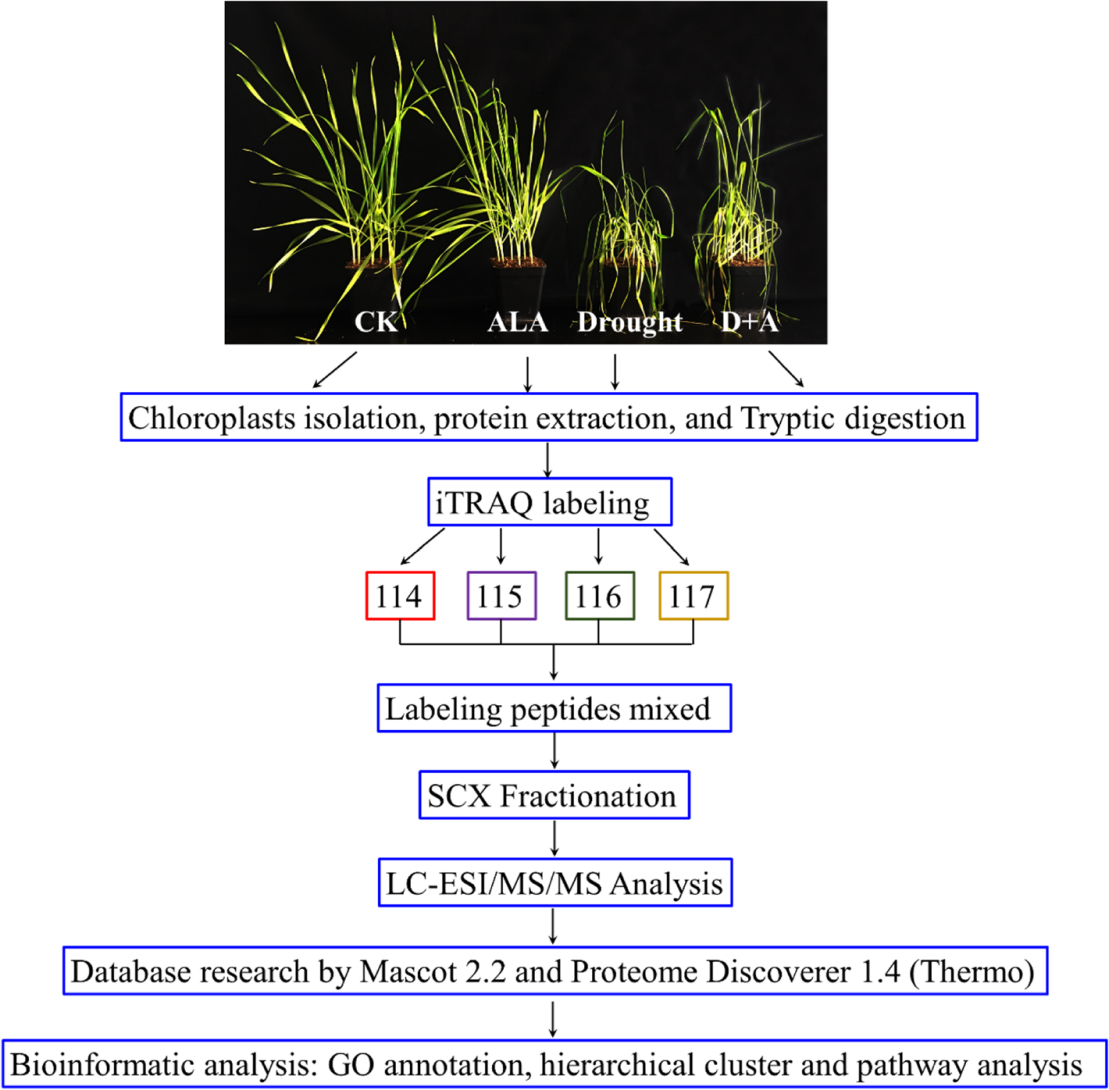
iTRAQ labeling and LC-MS/MS workflow to identify chloroplast proteins in wheat seedling leaves under drought stress and exogenous ALA pretreatment. CK, treated with 0 mg L^− 1^ ALA + distilled water; ALA, treated with 100 mg L^− 1^ ALA and distilled water; drought, treated with 0 mg L^− 1^ ALA without distilled water; D + A, treated with 100 mg L^− 1^ ALA without distilled water. The four treatments, CK, ALA, drought, and D + A, were labeled with iTRAQ tags 114, 115, 116, and 117, respectively

Seeds of the wheat cultivar Aikang-58 with uniform sizes were selected and surface sterilized using 5% H_2_O_2_ for 3 min. Aikang-58 was commercially acquired from Henan New Agricultural Seed Industry co., LTD. The wheat cultivar was selected as the material because it is a drought-tolerant cultivar that has been planted in a large area in northern China. After 12 h of being immersed in tap water, the identical and just germinated seeds were subjected to a subsequent 48 h of pregermination on a double layer of moist filter paper. The identical and germinated seeds were then transferred to a plastic flower pot (length 6.8 cm, width 6.8 cm, and height 7.1 cm) with 90 g of nutrient soil. The growth chamber was manually controlled under a 14 h/10 h (light/dark) photoperiod at 300 μmol m^− 2^ s^− 1^ of photosynthetic photon flux density (PPFD) provided by fluorescent lamps. The light period was set from 7 AM to 9 PM every day. The ambient environmental temperature and relative humidity were controlled at 25 °C/22 °C (day/night) and 75~85%, respectively. The wheat seedlings were watered with 400 mL of water every 2 d until two leaves were fully expanded, and 100 mg L^− 1^ ALA was foliar applied according to our previous evaluation results [23]. Three days after ALA treatments, the seedlings were subjected to drought stress by a cessation of watering, while the control group was watered as normal. The treatment combinations were set as follows: (1) CK, 0 mg L^− 1^ ALA + distilled water; (2) ALA, 100 mg L^− 1^ ALA + distilled water; (3) drought, 0 mg L^− 1^ ALA + without distilled water; (4) D + A, 100 mg L^− 1^ ALA + without distilled water. The culture soil was a mixture of organic nutrient soil and vermiculite ratio of 3:1 (v/v), which has a water loss rate of about 10.5% per day without water supplement. After 6 d of treatment, the second leaves (leaves immediately below the flag leaves) were collected for chloroplast isolation and proteomic analysis. On this time point, the water content of soil was ranging from 70 to 75% under normal watering condition, while it was ranging from 30 to 35% under drought condition. With the purpose of minimizing the deviation between different parallel samples under the same treatment, the sample was retrieved from independent three plots of wheat seedlings. The workflow is shown in Fig. 8.

### Physiological measurements

The stress parameter the wheat seedlings have suffered was evaluated using the soil water potential. The physiological traits were investigated including the leaf osmotic potential, relative water content (RWC), and chlorophyll content. Fresh seedling leaves were sampled from three replicates under each treatment. RWC was calculated by the following formula: RWC (%) = (FW – DW) / (TW – DW) × 100 [23]. The chlorophyll content was determined by spectrophotometry according to Porra et al. [66]. The leaf osmotic potential was measured using a water potential analyzer (WP4C, Decagon Devices Inc., USA).

### Chloroplast isolation

Pure and intact wheat chloroplasts were isolated from 10 g of fresh leaves according to Tamburino et al. with modifications [10]. All steps were carried out at 4 °C. Fresh leaves were homogenized thoroughly in 100 mL of phosphate buffer (pH 7.6) containing 330 mM D-sorbitol, 50 mM Hepes-KOH, 2 mM MgCl_2_, 2 mM EDTA-Na_2_, and 5 mM ascorbic acid. Homogenates were filtered through 4 layers of filter paper and washed until no green liquid overflowed. The filtrate was centrifuged at 300×*g* for 1 min, and the supernatant was centrifuged again at 1000×*g* for 2 min. Pellets were resuspended in phosphate buffer (pH 7.6) containing 330 mM D-sorbitol, 50 mM Hepes-KOH, and 1 mM DTT. The suspension was carefully loaded onto a contiguous Percoll gradient (Sigma-Aldrich, USA) and centrifuged at 10500×*g* for 10 min. Intact chloroplasts were carefully recovered and resuspended in a 5-fold volume of buffer containing 20 mM Tricine-KOH (pH 7.6), 5 mM MgCl_2_, 2.5 mM EDTA, and 0.3 M sorbitol. Organelle purity and intactness were monitored via fluorescence microscopy (Supplementary Figure 3). After centrifugation at 2100×*g* for 5 min, the pellets were recovered, quickly frozen in liquid nitrogen, and then stored at − 80 °C until use. Three biological replicates were used for each treatment condition.

### Chloroplast protein extraction and digestion

The chloroplast protein extraction procedure followed the instructions of the Sigma kit for the isolation of intact chloroplasts from leaves (Sigma, CPISO-1KT). The intact chloroplasts were solubilized in 1/10 volumes of SDT buffer (4% SDS, 100 mM DTT, and 150 mM Tris–HCl, pH 8.0). After 3 min of incubation in boiling water, the suspensions were ultrasonicated (80 w, 10 s ultrasonic at a time, every 15 s, and 10 times) and incubated at 100 °C for 3 min. The crude extract was clarified by centrifugation at 13,000×*g* at 25 °C for 10 min [67]. The extracted chloroplast proteins were quantitatively determined using the Bradford assay with BCA as a standard, and 20 μg of sample was used for the quality verification by an SDS-PAGE analysis. The supernatants were stored at − 80 °C until use.

Protein digestion was performed according to the filter-aided sample preparation (FASP) procedure described by Wisniewski et al. [67]. Two hundred micrograms of each extracted protein was added to 200 μL of UA buffer (pH 8.0) containing 8 M urea and 150 mM Tris-HCl. The mixture was transferred to a 10 kDa ultrafilter centrifuge tube and centrifuged twice at 14,000×*g* for 15 min. The sediment was dissolved in 100 μL of 50 mM IAA (prepared by UA buffer) and was shaken at 600 rpm for 1 min. After 30 min of incubation in the dark at room temperature, the dissolution was centrifuged at 14,000×*g* for 10 min. The sediment was again washed twice using 100 μL of UA buffer and then twice washed using 100 μL of dissolution buffer (50 mM triethylammonium bicarbonate, pH 8.5) by centrifuging the sample at 14,000×*g* for 10 min. Then, the proteins were digested in 40 μL of trypsin buffer, which contained 2 μg of trypsin in 40 μL of dissolution buffer. The digestion of the chloroplast proteins was performed at 37 °C for 18 h with shaking. The digested peptides were collected by centrifugation at 14,000×*g* for 10 min, and the sediment was discarded.

### iTRAQ labeling and SCX separation

Eighty micrograms of the peptides were labeled using the iTRAQ Reagent-4Plex Multiplex Kit (AB SCIEX) according to the manufacturer’s protocol. The four treatments, CK, ALA, drought, and D + A, were labeled with iTRAQ tags 114, 115, 116, and 117, respectively. The separation of the iTRAQ-labeled peptides by SCX was performed on an AKTA Purifier 100 (GE Healthcare). The iTRAQ-labeled peptides that were prepared as above were mixed, and 10× volume buffer A (10 mM KH_2_PO4, 25% v/v ACN, pH was adjusted to 3.0 with phosphoric acid) was added prior to loading the samples onto a polysulfoethyl 4.6 × 100 mm SCX 200 Å column containing 5 μm particles (PolyLC Inc., Maryland, U.S.A.). The peptides were eluted at a flow rate of 1 ml/min with a gradient of 100% buffer A for 25 and 25.01 min, 90% buffer A and 10% buffer B (10 mM KH_2_PO_4_ pH 3.0, 500 mM KCl, 25% CAN) for 32 and 32.01 min, 80% buffer A and 20% buffer B for 42 and 42.01 min, 55% buffer A and 45% buffer B for 47 and 47.01 min, 100% buffer B for 52 and 60 min, and 100% buffer A for 60.01 and 75 min. Then, in total, 33 fractions were freeze-dried and desalinized by a C_18_ cartridge prior to liquid chromatography-mass spectrometry (LC-MS) analysis.

### LC-MS analysis

The peptides of each fraction (10 μL autoinjections) were separated using an EASY-nLC1000 HPLC system (Thermo Scientific, USA) with a flow rate of 250 nL/min. The injection column was EASY-column C18 packed with 5 μm C18 particles (2 cm × 100 μm, Thermo Scientific, Waltham, USA), and the separation column was EASY-column C18 packed with 3 μm C18 particles (75 μm × 100 mm, Thermo Scientific, USA). The column was prebalanced by 95% solvent A (water, 0.1% v/v formic acid) and 5% solvent B (water, 0.1% v/v formic acid and 84% acetonitrile). Peptides were eluted by the application of a linear gradient mobile phase: a gradient from 0% solvent B to 35% solvent B over 55 min, followed by 35% solvent B to 100% solvent B over 3 min, which was maintained for 2 min.

A Q-Exactive system (Thermo Finnigan, USA) was applied in the MS analysis of the fractions separated by capillary HPLC. Data were acquired in the positive ESI mode with auto MS/MS in the range of m/z 300–1800. The parameters of the first level of MS were as follows: 70,000 at m/z 200 of the resolution level, 3e6 of AGC target, 10 ms of maximum IT, and 40 s of dynamic exclusion. The parameters of the second level of MS were as follows: 2 m/z of isolation window, 17,500 at m/z 200 of resolution level, 60 ms of maximum IT, 30 eV of normalized collision energy, 0.1% of underfill ratio, and the activation type was HCD.

### Bioinformatic analysis

The raw data files of the MS analysis were submitted to the Mascot 2.2 server (Matrix Science, Boston, USA) for relative quantification and protein identification using Proteome Discoverer software (version 1.4; Thermo, USA). The database used for the MS/MS data searching was the UniProt *Triticum aestivum* protein database, including 145,635 sequences that were released on July 17, 2017. The false discovery rate (FDR) was calculated and used to filter the effective peptide by the standard of FDR ≤ 0.01. The detected protein threshold in the software was set to achieve 95% confidence. The chloroplast proteomic analysis was performed with biological triplicates, and Student’s *t*-test was used to evaluate the significance of the DACPs between treatments at the level of *P* < 0.05. The iTRAQ ratios between different treatments higher than 1.5 and lower than 0.667 were selected to classify the proteins as up- or downregulated, respectively. PCA was carried out to assess the similarity and difference of chloroplast proteome among the four samples using online visualizing clustering software of ClustVis (https://biit.cs.ut.ee/clustvis/) [68].

The DACPs were functionally categorized according to the Gene Ontology (GO) annotation by BLAST2GO software (https://www.blast2go.com/). The pathways of the DAPs were predicted using the Kyoto Encyclopedia of Genes and Genomes (KEGG) (http://www.kegg.jp/kegg/). The cluster analysis of the DACPs was performed using Cluster 3.0 software (http://bonsai.hgc.jp/~mdehoon/software/cluster/software.htm). Protein–protein interactions (PPIs) were predicted by the Search Tool of the Retrieval of Interaction Genes/Proteins (STRING) database (Version 11.0) [69], and the interaction network was illustrated by Cytoscape software [70]. The PPIs with interaction scores higher than 0.7 (high confidence) were subjected to further interaction network analysis.

### RNA extraction and real-time PCR analysis

Total RNA was extracted from the second leaves and reversely transcribed to cDNA. The cDNA was used for quantitative real-time PCR analysis as previously described [23]. We randomly selected the genes for six differentially accumulated proteins to investigate their relative transcription levels. Primer pairs for qRT-PCR analysis were designed from the corresponding genomic sequences of the targeted proteins using the Primer 5 software (PREMIER Biosoft International, USA). The amplification of β-*actin* was used as an internal control [71]. All the primer sets were listed in Supplementary Table 3. The relative expression levels of specific genes were calculated using the 2^-ΔΔCT^ method [72].

### Determination of H_2_O_2_ content, O_2_^−^ production rate, and peroxidase (POD) and glutathione peroxidase (GPX) activities in the chloroplasts

The H_2_O_2_, content, O_2_^−^ production rate, and POD (EC 1.11.1.7) and GPX (EC 1.11.1.9) activities in the chloroplasts were determined according to the methods of Li et al. [73] immediately after the chloroplasts were isolated. The H_2_O_2_ content in the chloroplasts was determined by monitoring the absorbance of the titanium peroxide complex at 410 nm, and the production rate of O_2_^−^ was measured at an absorbance at 530 nm. The POD activity was determined by recording the changes at 470 nm during guaiacol oxidation for 4 min, and one enzymatic unit was defined as the change in 1 unit of absorbance per minute. The GPX activity was measured by monitoring the increase in the absorbance at 470 nm due to the oxidation of guaiacol.

### Data analysis

Three independent replicates were performed for each measurement, and the results are shown as the mean ± standard deviation (SD). GraphPad Prism 7.0 software (GraphPad software Inc., California, USA) was used for statistical analysis by one-way ANOVA, and the significant differences of the means among treatments were compared using Tukey’s multiple comparisons test at a level of *P* < 0.01.

## Supplementary information

**Additional file 1: Supplementary Table 1.** The identified peptides in chloroplast extracted from wheat leaves under different treatments.

**Additional file 2: Supplementary Table 2.** The identified proteins in chloroplast extracted from wheat leaves under different treatments.

**Additional file 3: Supplementary Table 3**. Primers in the real-time PCR assay.

**Additional file 4: Supplementary Figure 1.** Principal component analysis of chloroplast proteome of wheat seedling leaves under drought stress and exogenous ALA pretreatment. CK, treated with 0 mg L^− 1^ ALA + distilled water; ALA, treated with 100 mg L^− 1^ ALA and distilled water; drought, treated with 0 mg L^− 1^ ALA without distilled water; D + A, treated with 100 mg L^− 1^ ALA without distilled water. Vector scaling is applied to rows; Nipals PCA is used to calculate principal components. X and Y axis show principal component 1 and principal component 2 that explain 44.4 and 20% of the total variance, respectively. Prediction ellipses are such that with probability 0.95, a new observation from the same group will fall inside the ellipse. *N* = 12 data points including three independent biological replicates for each treatment.

**Additional file 5: Supplementary Figure 2**. The correlation of qRT-PCR and proteomic analysis for randomly selected DACPs.

**Additional file 6: Supplementary Figure 3.** The representative fluorescence microscopy images of extracted chloroplast. The chloroplasts were extracted from plants treated with distilled water (a), 100 mg L^− 1^ ALA and distilled water (b), 0 mg L^− 1^ ALA without distilled water (c), and 100 mg L^− 1^ ALA without distilled water (d). The wheat plants underwent drought stress 3 d after pretreatment with or without ALA.

## Data Availability

The datasets supporting the conclusions of this article are included within the article and its additional files. The mass spectrometry proteomic data of this article have been deposited to the ProteomeXchange Consortium via the iProX (Integrated Proteome resources) partner repository with the dataset identifier PXD017528.

## Abbreviations

ALA: 5-Aminolevulinic acid
CK: Control
DACPs: Differentially abundant chloroplast proteins
FASP: Filter-aided sample preparation
FDR: False discovery rate
GO: Gene ontology
GPX: Glutathione peroxidase
LC-MS: Liquid chromatograph mass spectrometer
POD: Peroxidase
PPIs: Protein–protein interactions
ROS: Reactive oxygen species
