# Supplementary material for: The iTRAQ-based chloroplast proteomic analysis of Triticum aestivum L. leaves subjected to drought stress and 5-aminolevulinic acid alleviation reveals several proteins involved in the protection of photosynthesis
Source: BMC Plant Biol. 2020 Mar 4;20:96. doi: 10.1186/s12870-020-2297-6 (PMC7057492; doi:10.1186/s12870-020-2297-6)
Supplement: Supplementary file 3 — Additional file 3: Supplementary Table 3. Primers in the real-time PCR assay. [file 12870_2020_2297_MOESM3_ESM.docx]

**Supplementary Table 3**. Primers in the real-time PCR assay.

| Target name | Primer name | Sequence (5’ → 3’) |
| --- | --- | --- |
| A0A1D5YCF1 | A0A1D5YCF1-Forward | GAGGAGGGAGTCCTACTGGT |
|  | A0A1D5YCF1-Reverse | AGCAGCAACCTCCTTGATCT |
| Q8S9H0 | Q8S9H0-Forward | CCTACGAGCTCATGGAGGAC |
|  | Q8S9H0-Reverse | CGGGACGTAGAACTTGACC |
| A0A1D5WTV3 | A0A1D5WTV3-Forward | CGCGTCTGACATGTAACTGG |
|  | A0A1D5WTV3-Reverse | GCAAGTCCATACAGCGCAAA |
| A0A1D6BSD3 | A0A1D6BSD3-Forward | GGTCGTCTTGCCATGTTCTC |
|  | A0A1D6BSD3-Reverse | ACGAAGTTAGTGGCGAATGC |
| A0A078BQY4 | A0A078BQY4-Forward | TCTACATCGCTCCTGCTTTCAT |
|  | A0A078BQY4-Reverse | AGCTCGCACTGGAATGATTTT |
| A0A078BTE9 | A0A078BTE9-Forward | GAGGCTGCCGACATTATCAA |
|  | A0A078BTE9-Reverse | GTTGTTCACCGTGTACTGCGT |
| Actin | Actin-Forward | AGCGGTCGAACAACTGGTA |
|  | Actin-Reverse | AAACGAAGGATAGCATGAGGAAGC |
