# Supplementary figures and images for: The iTRAQ-based chloroplast proteomic analysis of Triticum aestivum L. leaves subjected to drought stress and 5-aminolevulinic acid alleviation reveals several proteins involved in the protection of photosynthesis
Source: BMC Plant Biol. 2020 Mar 4;20:96. doi: 10.1186/s12870-020-2297-6 (PMC7057492; doi:10.1186/s12870-020-2297-6)

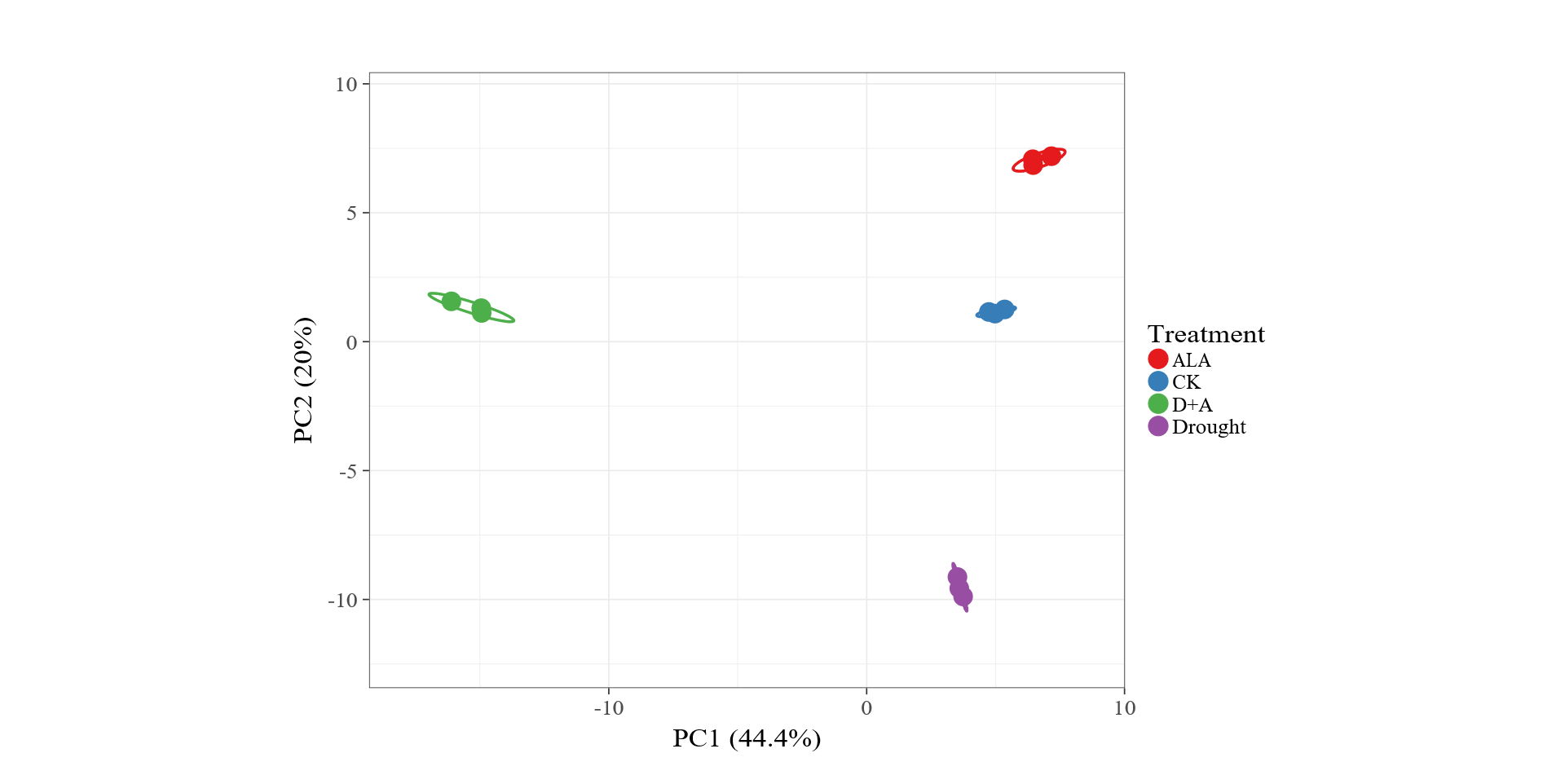


**Supplementary Figure 1.**

Supplement: Supplementary file 4 — Additional file 4: Supplementary Figure 1. Principal component analysis of chloroplast proteome of wheat seedling leaves under drought stress and exogenous ALA pretreatment. CK, treated with 0 mg L− 1 ALA + distilled water; ALA, treated with 100 mg L− 1 ALA and distilled water; drought, treated with 0 mg L− 1 ALA without distilled water; D + A, treated with 100 mg L− 1 ALA without distilled water. Vector scaling is applied to rows; Nipals PCA is used to calculate principal components. X and Y axis show principal component 1 and principal component 2 that explain 44.4 and 20% of the total variance, respectively. Prediction ellipses are such that with probability 0.95, a new observation from the same group will fall inside the ellipse. N = 12 data points including three independent biological replicates for each treatment. [file 12870_2020_2297_MOESM4_ESM.docx]

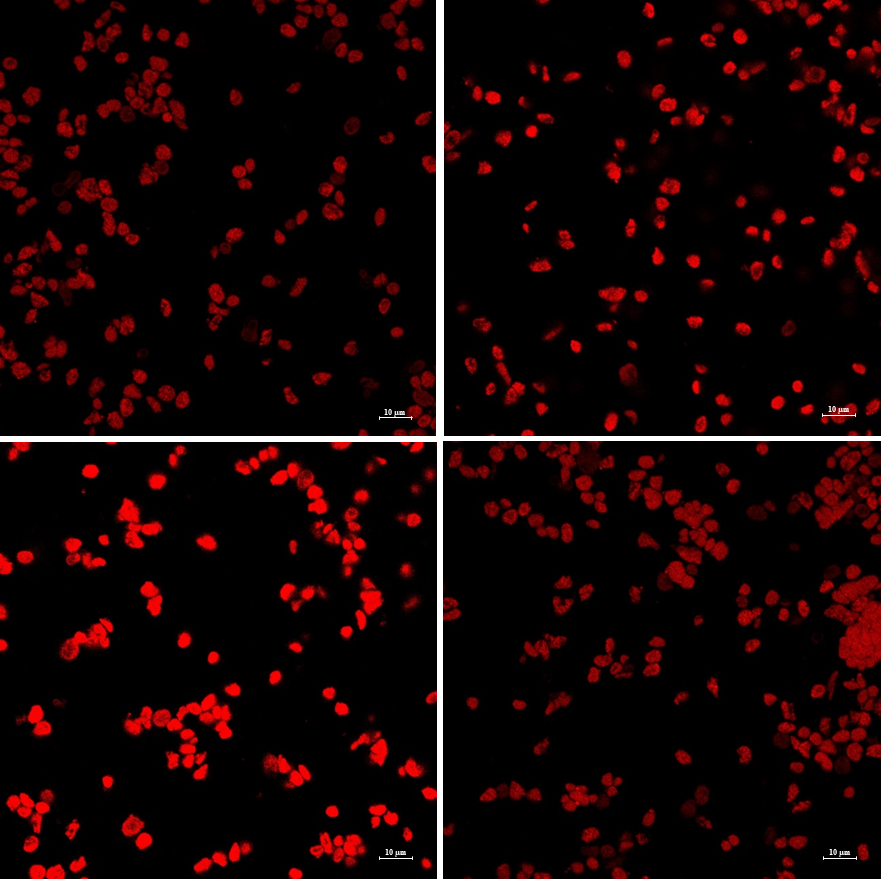


a

b

c

d

**Supplementary Figure 3.**

Supplement: Supplementary file 6 — Additional file 6: Supplementary Figure 3. The representative fluorescence microscopy images of extracted chloroplast. The chloroplasts were extracted from plants treated with distilled water (a), 100 mg L− 1 ALA and distilled water (b), 0 mg L− 1 ALA without distilled water (c), and 100 mg L− 1 ALA without distilled water (d). The wheat plants underwent drought stress 3 d after pretreatment with or without ALA. [file 12870_2020_2297_MOESM6_ESM.docx]
